# Augmented Reality for the Assessment of Children's Spatial Memory in Real Settings

**DOI:** 10.1371/journal.pone.0113751

**Published:** 2014-12-01

**Authors:** M.-Carmen Juan, Magdalena Mendez-Lopez, Elena Perez-Hernandez, Sergio Albiol-Perez

**Affiliations:** 1 Instituto Universitario de Automática e Informática Industrial, Universitat Politècnica de València, Camino de Vera, s/n. 46022 Valencia, Spain; 2 Departamento de Psicologia y Sociologia, Universidad de Zaragoza, Zaragoza, Spain; 3 Departamento de Psicologia Evolutiva y de la Educacion, Universidad Autonoma de Madrid, Madrid, Spain; 4 Departamento de Informatica e Ingenieria de Sistemas, Universidad de Zaragoza, Zaragoza, Spain; Brock University, Canada

## Abstract

Short-term memory can be defined as the capacity for holding a small amount of information in mind in an active state for a short period of time. Although some instruments have been developed to study spatial short-term memory in real environments, there are no instruments that are specifically designed to assess visuospatial short-term memory in an attractive way to children. In this paper, we present the ARSM (Augmented Reality Spatial Memory) task, the first Augmented Reality task that involves a user's movement to assess spatial short-term memory in healthy children. The experimental procedure of the ARSM task was designed to assess the children's skill to retain visuospatial information. They were individually asked to remember the real place where augmented reality objects were located. The children (N = 76) were divided into two groups: preschool (5–6 year olds) and primary school (7–8 year olds). We found a significant improvement in ARSM task performance in the older group. The correlations between scores for the ARSM task and traditional procedures were significant. These traditional procedures were the Dot Matrix subtest for the assessment of visuospatial short-term memory of the computerized AWMA-2 battery and a parent's questionnaire about a child's everyday spatial memory. Hence, we suggest that the ARSM task has high verisimilitude with spatial short-term memory skills in real life. In addition, we evaluated the ARSM task's usability and perceived satisfaction. The study revealed that the younger children were more satisfied with the ARSM task. This novel instrument could be useful in detecting visuospatial short-term difficulties that affect specific developmental navigational disorders and/or school academic achievement.

## Introduction

Memory is a cognitive process that is necessary for the stable acquisition of skills or information. This process is crucial for the appropriate learning of any behaviour. The processes of memory can be classified according to their duration in sensory memory, short-term memory, and long-term memory. Sensory memory corresponds to approximately the initial 200–500 milliseconds after an item is perceived. The ability to look at an item and remember what it looked like with just a second of observation or memorization is an example of sensory memory. Short-term memory allows recall for a period of several seconds to a minute without rehearsal. In contrast, long-term memory can store much larger quantities of information for a potentially unlimited duration. The classification of memory based on the temporal extension of the information memorized could also be combined with the type of material to be retained. Thus, spatial memory generally refers to the ability to generate, represent, transform, and recall spatial information [1]. In other words, spatial memory is a cognitive process that enables a person to remember different locations as well as spatial relations between objects. Consequently, it can also be divided into spatial short-term memory and spatial long-term memory. Spatial short-term memory can be described as a system that allows us to temporarily store and manage spatial locations. This allows us to remember where an object is in relation to another object. However, spatial long-term memory can store much larger spatial information for a potentially unlimited duration. The spatial short-term memory is necessary to be able to complete complex cognitive tasks such as those related to aspects of mathematics, especially with number writing and magnitude judgment [2]. This kind of memory also predicts learning outcomes at school [3]. The spatial short term memory can be affected in children with specific language impairment, a persistent disorder that has a negative impact on academic performance [4]. However, the learning difficulties that are more clearly associated with spatial short-term memory impairment are dyscalculia [5] and non-verbal learning disabilities [6]. Some of these studies have examined groups of children using the Automated Working Memory Assessment (AWMA, [7]), which includes subtests for the assessment of spatial short-term memory capacity. Thus, spatial short-term memory is closely related to academic skills and has implications for children's school performance.

There is great interest in the field of human memory, its properties, and neural substrates. However, most of the research in this field has focused on experimental paradigms for the assessment of spatial memory in rodents. The main reason for the significant amount of leading research with rodents is the ethical problems that are derived from research that attempts to understand the human brain circuits involved in memory. Therefore, researchers have used the innate ability of rodents to remember places in order to carry out their studies. Spatial memory tasks adapted for humans involve the simulation of movement through the space by stationary subjects. However, the everyday skills required in memory for locations involves a person's movement through the environment, and the reduced ecological validity of these conventional tasks could be overcome with a tool that combines a strong control of stimuli presentation in real settings.

In this paper, we present ARSM (Augmented Reality Spatial Memory) task, the first Augmented Reality (AR) task that assesses spatial short-term memory in children involving movement. The objective of the study was to prove the value of the ARSM task in assessing spatial short-term memory by comparing the children's performance for the developed task with current approaches for testing spatial short-term memory. The primary hypothesis was that the results for the ARSM task would reflect the spatial short-term memory ability of children in the same way as traditional procedures. In addition, the results obtained with this new procedure would have a relationship with spatial memory performance in everyday life.

The paper is organized as follows. Section 2 focuses on the state of short-term memory and AR applications related to learning. Section 3 details a preliminary study that was carried out to determine the most appropriate size of the device for the ARSM task. Section 4 describes the task and briefly explains the software and hardware required to develop and run the ARSM task. Sections 5, 6, and 7 present the study, the results, and the discussion, respectively. Finally, in Section 8, a number of conclusions and areas for future research are identified.

## Background

### Short-term memory

Both long-term and short-term memory have traditionally been assessed using animals [8]–[12]. Laboratory animals have been trained in mazes in which they have to remember spatial information that can be in long-term or short-term storage. The increasing knowledge of Virtual Reality (VR) techniques and the tradition of rodent research in spatial memory have led to the development of VR-based mazes for humans that reproduce the demands of tasks previously used for rodents [13]–[19]. Those VR systems were quite basic; they used common monitors, very basic interaction methods (such as mouse-clicking or a joystick), and, more importantly, without using movement. For example, Kelly and Gibson [16] examined the use of featural and geometric information in adults by having them navigate in a virtual environment that was designed to be similar to the real-world environment experienced by rats [20]. Men and women were trained to locate an element in one of four corners of a fully enclosed rectangular room. The interaction with the virtual environment was done by mouse-clicking. The geometric area task was initially developed by Cheng [20] to examine whether rats could encode featural and geometric properties of the environment.

Astur et al. [14], [15] developed virtual navigation software for the assessment of human spatial memory, especially in psychiatric groups with brain abnormalities such as schizophrenia, epilepsy, or alcohol intoxication. They designed a human version of the Water Maze task called the Virtual Reality Pool task. Using a virtual environment on a conventional monitor, the subjects were placed in a circular pool in a room with distal cues. The task consisted of a virtual environment in which the subjects used a joystick to escape from the water as quickly as possible by reaching a hidden platform under the surface of the water. Since the platform was not visible, a good performance for this task depended on the spatial memory recall of the integrity of the distal cues presented. Also, using the same technological approach, they developed the Virtual Eight-Arm Radial Maze. In this maze, the subjects found themselves in a virtual room that had eight runways extending out from a circular central area. There were rewards at the end of four of the runways and the subjects had to determine their location as soon as possible. Similarly, Cánovas et al. [18] developed the “*Boxes Room*” task. The subjects were asked to locate the boxes with rewards in a computer-generated environment with sixteen boxes. The position of the hidden elements was determined in relation to intra-maze or extra-maze cues. The intra-maze condition was composed of three columns of different colors placed between the boxes and there were no decorations or pictures on walls. In the extra-maze condition the room walls had various marks that disambiguated spatial locations including a window, a door, and pictures. They also designed an environment based on the “*active place avoidance*” task. The task was to virtually navigate through a circular room by manipulating a joystick. The aim was to avoid an unmarked place while collecting rewards in the arena.

For tasks that are not based on paradigms for rodents, Burgess et al. [21], [22] used VR environments that are based on modifications of video games to study the neural basis of episodic and spatial memory. Mainly VR towns were used, in which the subject's movements were generated using a keypad or joystick. These towns consisted of several buildings and roads through which subjects could move. The subjects were trained to find their way around the town. They practiced following a route, of arrows, meeting a person on the route and getting several objects. In addition, Koening et al. [23] developed the Virtual Memory Task (VMT) for cognitive rehabilitation of patients with brain injury. The VMT is especially good at increasing awareness of cognitive deficits in brain injury patients. In that case, the novelty of the task resided in the personalization of the virtual environment in relation to the real environment. That is, the task was implemented in a virtual model of the office room inside a clinic in which they were seated during the testing session. The VMT had sufficient details and photorealistic textures so that it was easily recognized by the participants. The VMT was displayed on a monitor placed in front of the participants. A keyboard and mouse were used to interact during the task. The participants were instructed to memorize the locations of typical office objects that were placed on a table. After this, a different view was presented in which the objects were moved to new locations and the participants were asked to move the objects back to the initial location. In this new view, the perspective also changed. This change caused confusion whenever the virtual perspective was different from the real perspective.

Little attention has been paid to the ecological validity of tasks for the assessment of memory and especially for spatial short-term memory. Although VR has improved this issue with the presentation of naturalistic stimuli, there still exist some problems. Studies that have focused on spatial short-term memory load have not yet taken into account the person's performance in real-world settings. In addition, only 2-D tasks have commonly been used. The short-term memory task of Passolunghi consisted of a recall of positions occupied by stimuli on a grid that appeared on a computer screen [24]. The responses were given using the mouse. In the study by Thomas, children were tested on a search task of a computerized hidden pathway maze using a touch screen. The pathway was concealed in a 2-D tile grid [25]. However, in their study, Spooner and Pachana [26] suggested that the verisimilitude in tasks with situations that children encounter every day is necessary because it increases the predictability for the children's functional mastery.

### Augmented Reality

AR is a technology that is currently being incorporated in many fields such as psychology [27] or education [28]. AR allows the user to see the real world, with virtual objects that are superimposed upon the real world to supplement reality. In an ideal AR application, the real and virtual objects would appear to coexist in the same space. Mobile devices with their current features are ideal for running AR applications anywhere and at any time. Thanks to these capabilities, several AR applications have already been developed and tested. Nevertheless, to our knowledge, AR has not been used to assess cognitive processes. One of the fields in which mobile AR systems have already proven their potential is the educational field. For example, Juan et al. [29] developed a mobile game using a Nokia N95 to raise individuals' awareness of the importance of recycling and teaching participants how to do it. They compared an AR game with a video game. The aspects that were examined included the level of engagement and fun of each game, the ease of use and perceived value of each game, and the perceived learning about recycling. They reported a positive change in intended behavior with both games. Furió et al. [28] developed a game for learning the water cycle. They compared two devices (an iPhone and a Tablet). From their results, they observed that the different characteristics (screen size and weight) of the devices did not influence the children's acquired knowledge, engagement, satisfaction, ease of use, or AR experience. Furió et al. [30] developed an iPhone game for learning multiculturalism, solidarity, and tolerance. For learning outcomes, their results did not show significant differences between the group that played with the iPhone game and the group that played traditional games. Albrecht et al. [31] compared a mobile AR system with textbook material for forensic medicine. Only 10 third-year medical students participated in the study. Their results showed a statistically significant increase in knowledge for the AR group. Liu and Tsai [32] presented mobile AR-based learning material in EFL English (English as a Foreign Language) composition. Only 5 participants took part in the study. The results showed that the participants were engaged in the learning scenario, constructed linguistic and content knowledge, and produced meaningful essays. In all these works, there is a common feature, which is to highlight the potential of AR, (especially mobile AR) for learning different types of contents or subjects, and the suggestion that this technology could be exploited in other fields. In this paper, we try to demonstrate that mobile AR also has great potential for assessing a cognitive process, specifically, spatial short-term memory.

### Preliminary study

A preliminary study was carried out to determine the most appropriate size of the device for the ARSM task to assess spatial short-term memory in children. Twenty-one preschool children (5 years old) participated in this study. Two of the children had special educational needs. The two devices used were a Galaxy Note mobile phone (5.5") and a Motorola tablet (8.2").

The procedure was the following. Two boxes were placed with a mark in its interior. The child had to go to the boxes from a starting point source located 1 meter away from the midpoint of the boxes. All the children used the two devices, but the sample was counterbalanced. The task for each child was to open one of the boxes and focus on the marker. At that moment, a 3D object appeared over the marker. Then with the other device, the child had to open the other box and look for the same object by focusing on another marker that was exactly the same as the one used in the first box. Once the task with the two devices was carried out, the children had to point to the device they liked the most.

From the results, the majority of children preferred the Motorola device (18 preferred the Motorola versus 3 who preferred the Galaxy Note). There were no differences between the times taken to complete the task based on the device used.

In addition to the conclusions obtained from the data, we also obtained the following conclusions. An external case for the two devices is recommended so that the children can easily hold them. Now the children hold the devices having to cover part of the screen, and, in most cases, the camera is also covered. The external case should be as light as possible to avoid making the weight of the device too heavy. With regard to the orientation, landscape is more intuitive and easier to handle than portrait.

## ARSM task

### Description of the ARSM task

The ARSM task is based on the multicomponent model of Baddeley [33]. According to this model, visuospatial short-term memory is conceived as a store with a limited capacity to retain visual and spatial information in terms of the number of items that must be considered. Short-term memory tests commonly determine the visuospatial memory span across several trials with different levels of difficulty depending on the number of elements to retain (from 1 to 10). The memory span of young adults is around seven elements. The locations are usually presented in a small matrix that is printed on a sheet of paper. The basic principle of the ARSM task is to show objects in a location and have the children remember where they were. The number of objects/locations to be retained increases throughout the task depending on the performance in previous trials.

**Figure 1:**
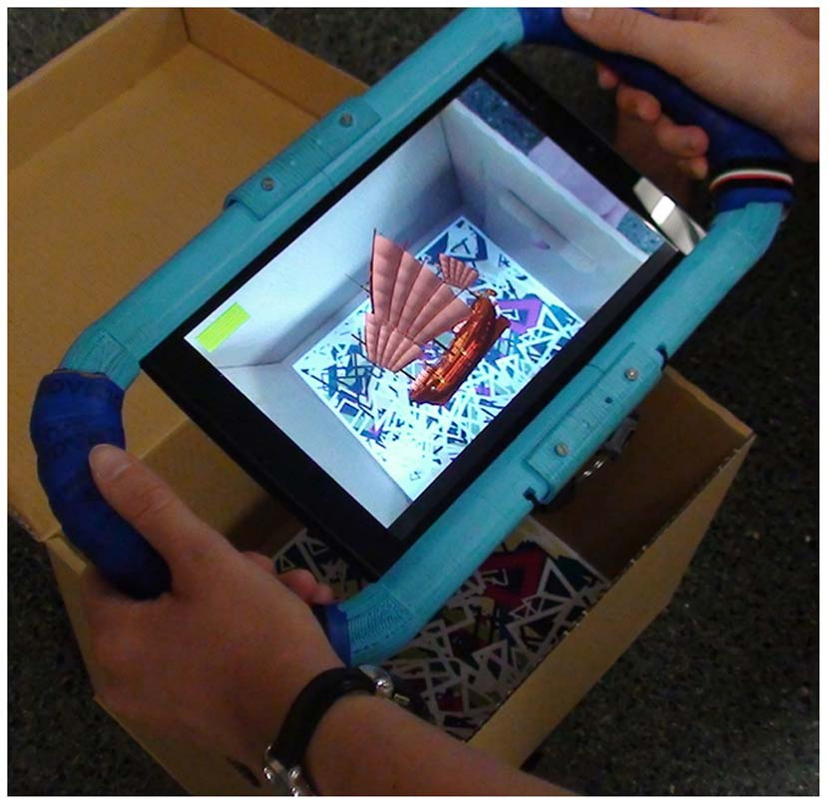
User playing with Block VI.

**Figure 2:**
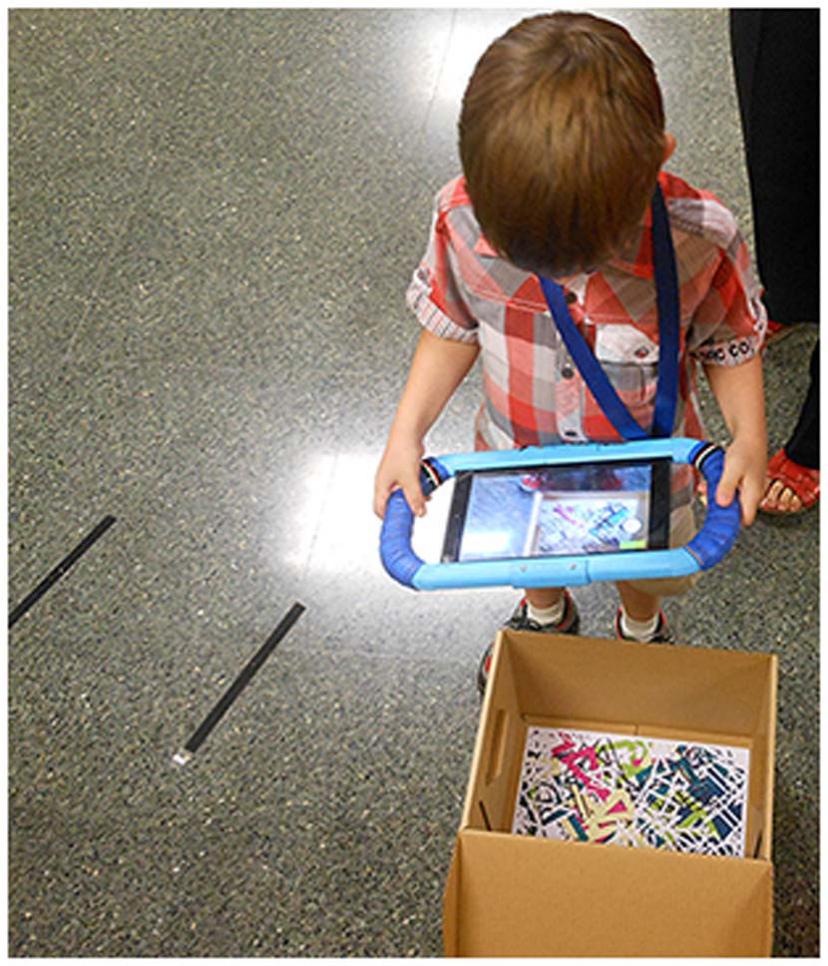
Child playing with Block II.

As an AR system, the objects are shown when the image targets are focused on with the camera from the Tablet. The image targets are placed inside boxes, which serve as locations, and these boxes are strategically located in the testing area. Figures 1 and 2 show two examples.

**Figure 3:**
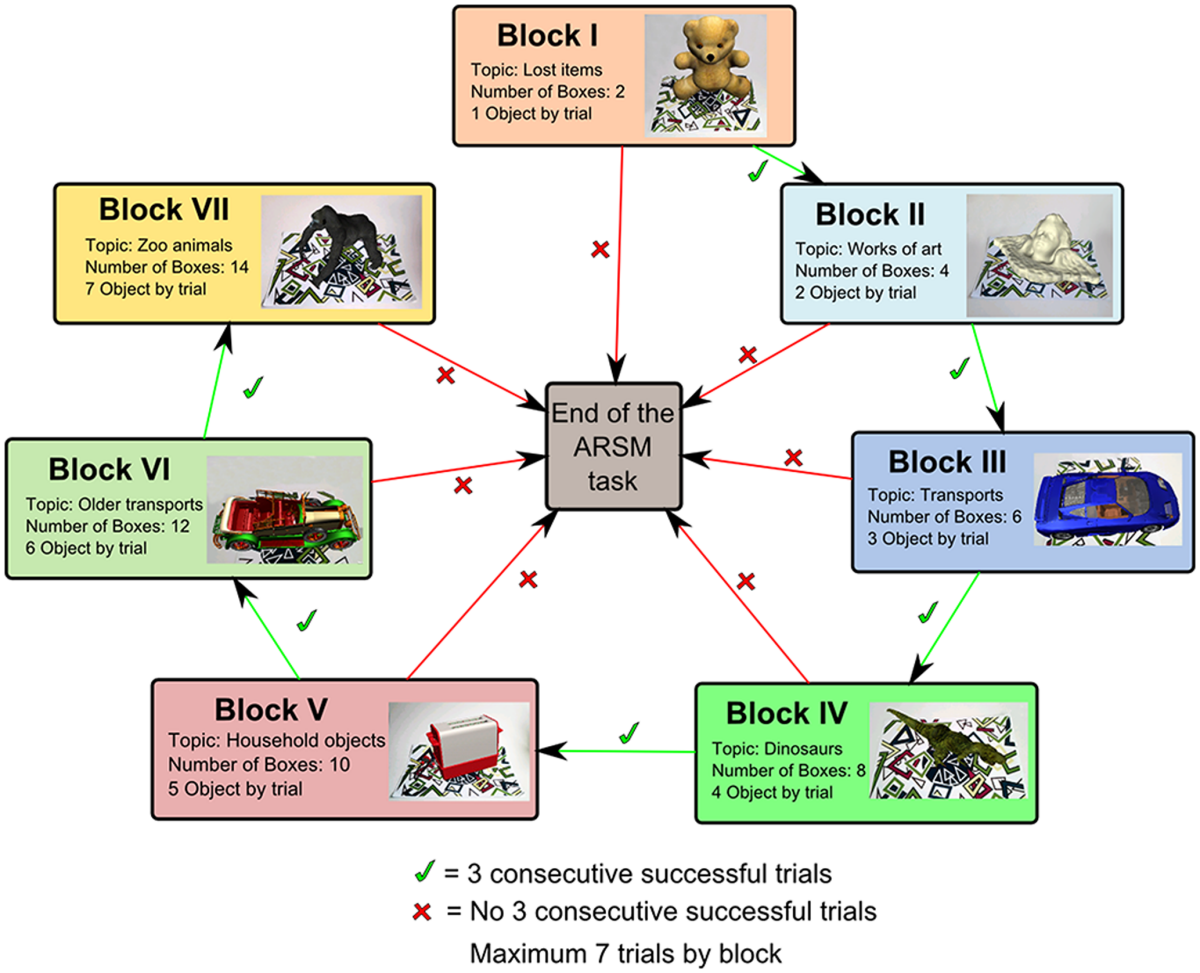
Flowchart of the ARSM task.

**Figure 4:**
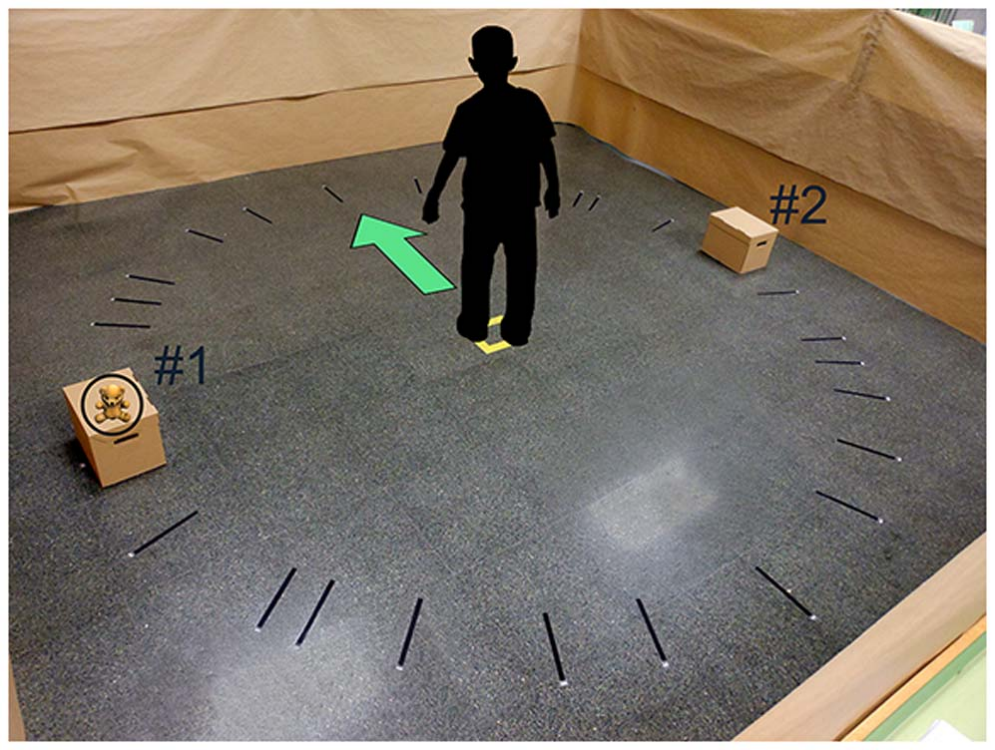
Scenario for the first block.

**Figure 5:**
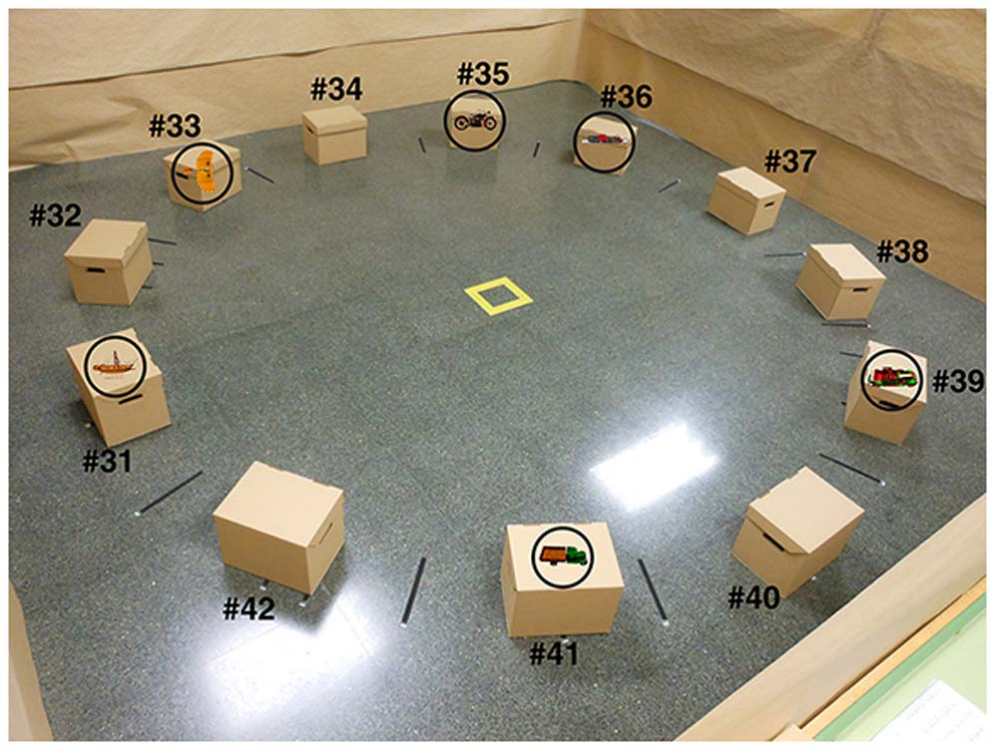
Scenario for the sixth block.

The ARSM task consists of seven different levels of Blocks, which are shown graphically in the flowchart in Figure 3. The maximum level is based on the memory span of young adults mentioned above. The difference among Blocks is the number of boxes (image targets) and the objects used in each trial. Each block contains a maximum of seven trials to ensure that performance is not a matter of chance. Each trial consists of two phases. In the first phase, which is called the search phase, the children have to search for the objects and remember the box (location) where they were. In the second phase, called the save phase, the ARSM task shows an object and the children have to remember the box where it was (successes or failures are counted). From a cognitive perspective, the search phase refers to the formation of short-term memories for visuospatial items, whereas the save phase refers to the retrieval of those items. The chances for completing a particular Block are determined by the number of successes and/or failures. If there are three consecutive successful trials for a number of trials less than or equal to seven, a block ends successfully. If trial V is reached and a fail is registered, the block ends unsuccessfully because it is not possible to have three more successes (only two could be achieved). A child goes to the next Block if he/she successfully passes the previous Block. The objects to be found in each trial are equal to the level of difficulty (block 1 – block 7) and the number of boxes is the level×2 so that the difficulty increases especially when the blocks have a low number of items (e.g. one or two). In the first Block there are two boxes and only one object to be found in each trial. In the second Block, there are four boxes and two objects to be found in each trial. In the third Block, there are six boxes and three objects to be found in each trial. The same sequence is followed for the rest of the Blocks. Figure 4 shows the scenario for the first Block, and Figure 5 shows the scenario for the sixth Block. No child successfully completed the sixth Block. To clarify the process, and as an example, the steps followed in the first Block are explained below. There are two boxes with two different image targets (#1 and #2 in Figure 4) and only one object appears in each trial. The starting position of the children is the center of the testing area and it is indicated as a yellow square (Figure 4). The children have to look to their left as shown in Figure 4.

- Trial I. In the first trial, the object appears over #1. At this moment, the child starts the search phase and can open either of the two boxes looking for an object. However, to reduce the time of the activity, the person in charge tells the child which box to look in. In this case, this person advises the child to open the box situated at his left. An object (a teddy bear) appears over #1. The child has to go to the starting position. The ARSM task asks the child to find the box where the teddy bear was. If the child goes to #1, the system registers a success. On the contrary, if the child goes to #2, the system registers a failure.
- Trial II. In the second trial, the object appears over #2. The procedure is exactly the same as Trial I. In the save phase, if the child goes to #2, the system registers a success, otherwise a failure is registered.
- Trial III-Trial VII. In these trials, the object appears over #2, #1, #1, #2, and #1, respectively. The procedures are exactly the same as in the previous trials. After trial 3, if the number of consecutive successful trials is three, the child goes to the next Block. If trial V is reached and a failure is registered, the Block ends unsuccessfully because it is not possible to have three more successful trials (only two could be achieved).

**Figure 6:**
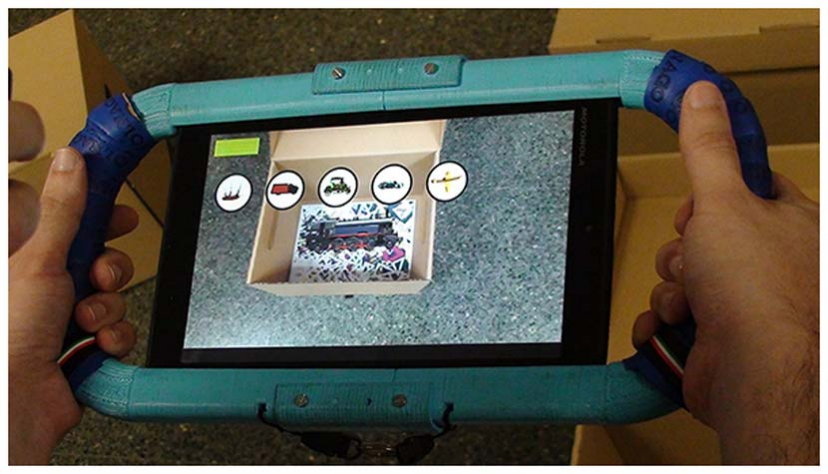
Design of the external case.

To keep the children motivated, the ARSM task includes a guide character, Mabu, and ‘mission’ messages. Mabu guides the children throughout the task. Mabu's purpose is to help the children focus on the task. At first, Mabu introduces herself and tells the children what they have to do (see the welcome message shown in Table 1). An arrow appears in the bottom-right area of the screen. The child has to touch the arrow to start the task. This method of interaction is the one that has been used throughout the ARSM task. That is, the guide character asks the children if they are ready, and when they are ready, they touch the arrow. The guide character can also tell the children to go to the starting point and then touch the arrow. The amount of information/number of elements that appear on the screen is kept to the minimum to facilitate the children's concentration. After the welcome message and when the child is ready, the guide character introduces the first mission for the first Block. This introduction is useful for the contextualization of the different blocks in order to make them attractive and challenging to the children. As an example, the audio for Block IV is shown in Table 1. When the child is ready to start the search phase, the child touches the arrow. The child opens a box and focuses on the image target. If an object is associated with this image target, the object appears. After 5 seconds, it disappears. Even if the child focuses on the image target, the object does not appear anymore. During the search phase, all the objects that have appeared in a trial are shown inside white circles in the upper-left area of the screen. Figure 6 shows an example. When the child is in the save phase, the device shows the object to search for and asks the child to look for it. An image of the object being searched for appears inside a white circle in the upper-left area of the screen. During the save phase, the guide character indicates whether the child has succeeded or failed in the selection of the box.

**Table 1:** Examples of messages given by the ARSM task.

Message	Content
**Welcome message**	Hi, I am Mabu. I am going to check how see you can remember clues. What you have to do is to hide clues in boxes and then you have to remember where you hid these clues. You have to pay close attention to pass a level and go to the next one. Are you ready? Then click the arrow.
**Introduction for Block IV**	Dinosaurs are in the wrong era! In this mission we have to take them back to the Jurassic period. To catch them you need to find their favorite tree and play some magical music. You have to open the boxes to see where each clue is. Watch them carefully for 5 seconds to remember which box each clue is in. Remember that to complete the mission you must find each of the clues. Are you in the starting position? Then click the arrow.

## Software and Hardware

We used the following software to develop the ARSM task:

1. Unity (also called Unity3D). Unity is a cross-platform game engine. It supports code written in C#, JavaScript, or Boo. Unity can read.fbx, dae (Collada), 3DS, dxf and.obj files.
2. Vuforia. SDK for the development of AR applications for Android and iOS. Vuforia SDK has an extension for Unity. It uses Computer Vision techniques to recognize and track different types of targets such as Image Targets (natural features), Frame Markers (particular type of 2D images), or Multi-Targets (simple 3D objects, rectangular shapes).
3. C#. We developed the ARSM task using C#.
4. The 3D models were treated with Autodesk 3DStudio 2009. Textures were treated with Adobe Photoshop. The ARSM task includes 196 3D models.
5. For the AR functionality, we designed 56 different image targets. At first, we designed 28 image targets by using Adobe Illustrator, and then, we rotated them horizontally and modified them to obtain 56 different image targets. The system can distinguish the images, but the images look very similar to the users who cannot distinguish the differences. Two of these image targets can be seen in Figure 1 and Figure 2.

To run the ARSM task, we used a Motorola Xoom 2 Media Edition with the following features: dimensions: 8.50" × 5.47" × 0.35"; weight: 13.62 oz; a TFT capacitive touchscreen display of 8.2"; a 5MP camera; and Android 4.x Operating System.

**Figure 7:**
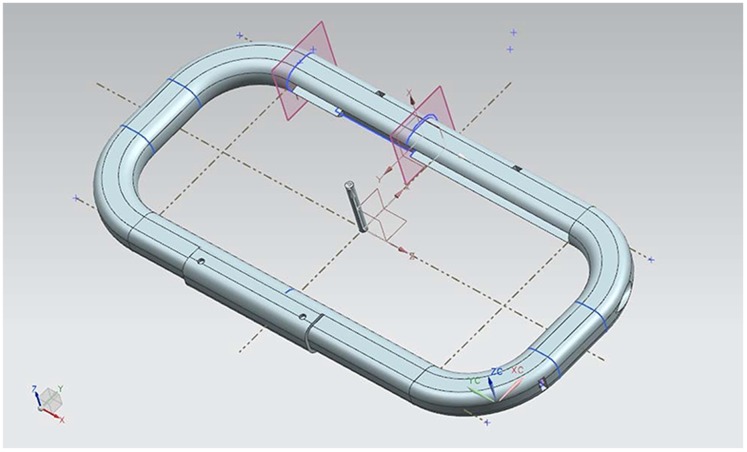
User playing with Block VI.

To protect the device from damage and also to provide more stability when holding the device, we designed and printed an external case on a 3D printer. For the external case to weigh as little as possible, the design only took into account the edges of the tablet. An outline that matches the edge of the tablet was generated based on the actual measurements of the tablet. This outline was spread along the perimeter, leaving room for the handles. Figure 6 shows the printed external case and Figure 7 shows the design. The 3D printer that we used was a Rapman 3.1. 3D printing is achieved using an additive process, where successive layers of material are laid down according to the design pattern. The material used was ABS white, which was then painted blue. Since the Rapman 3D printer cannot print elements of the size of the external case in one piece, the printing was divided into smaller pieces, which were subsequently joined by means of adhesive. The external case can be assembled and disassembled through the upper and lower central joints that are not glued and that are joined by two pieces that are bolted. To protect the device from damage and also to facilitate its handling, a ribbon was also added (Figure 2).

The size of the boxes used was: 26×35×25 cm. The place for testing was a square of about 5 meters on each side. It was surrounded by light brown paper to a height of 1.5 meters. This height was considered to be enough for an egocentric reference.

To distribute the boxes, a circle with a radius of 1.85 meters was used. The center of the circle was the position of the user (shown in Figures 4 and 5 as a small yellow square). The angle between boxes was defined by the number of boxes and was the same for all of them. To obtain this angle, the 360° of a circle are divided by the number of boxes. The first box in every block was numbered as # 1 (see Figure 4). For example, in the first Block, there were two boxes which were separated by 180°. For Block 6, the angle between boxes was 30°.

## Study

### Participants

**Figure 8:**
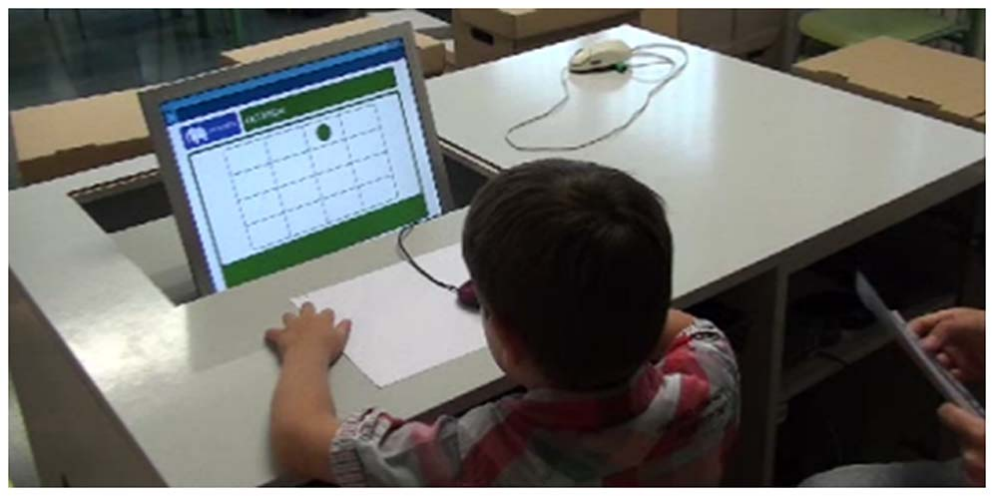
Child using the AWMA-2 Dot test.

Seventy-six healthy children, with ages between 5–8 years old, took part in the study. The mean age was 6.84 ± 1.12 years old. There were 41 boys and 35 girls. They were divided into two age groups: preschool (5–6 year olds, n = 41, 58.5% were boys and 41.5% were girls); and primary school (7–8 year olds, n = 35, 48.6% were boys and 51.4% were girls). Their parents received information about our study and they signed a consent form to allow their children to participate in it. All of the children verbally agreed to participate. The parents of the individual shown in Figures 2 and 8 of this manuscript have given written informed consent (as outlined in PLOS consent form) to publish these case details. Moreover, all clinical investigation was conducted according to the principles expressed in the Declaration of Helsinki. The Ethics Committee of the Technical University of Valencia approved this study. The data are available in Data S1. The participants received a small reward consisting of a diploma right after the testing sessions.

### Measurements

For each run of the ARSM task all the information required for the following variables is stored in a remote database: duration of the experiment, blocks completed, trials completed, total trials, % trials passed, errors, approximation errors, % approximation errors, % of errors, and score. The score represents the sum of the values obtained in each block according to the following: a value of 7 per block if 3 successes were obtained running the first three trials; a value of 1 per each successful trial for the remaining blocks.

After playing the ARSM task, the children answered a questionnaire composed of 9 questions, the ARquestionnaire. This questionnaire consisted of six questions about satisfaction and three questions about usability.

To compare the ARSM task with existing assessment procedures, the children were also tested using the two following tools:

1. The Dot Matrix subtest of the Automated Working Memory Assessment 2 (AWMA-2) [34]. We refer to it as AWMA Dot. The AWMA is an automated, computerized assessment battery that assesses different components of working memory. The AWMA Dot assesses the children's visuospatial short-term memory and is administered on a computer. In this subtest, a sequence of red dots is presented on a 4×5 grid. In our study, all of the dots appeared on the grid for 2 seconds. The dots then disappeared and the child had to point to the position of each dot in the same serial order as presented. The mouse was used for pointing out the positions (Figure 8).
2. The Parent questionnaire of the *Evaluación Clínica de la Memoria* (ECM-Q). This questionnaire is completed by the parents and consists of questions about their children's everyday memory. For our study, we selected eight Spatial Memory items [35]. The questions are related not only to spatial short-term memory, but also to long-term memory. The parents rated their child's skill on a 4-point Likert scale (1  =  *never* to 4  =  *almost always*). The questions are "My child has good spatial orientation, gets lost where he/she has often been before, forgets where he/she has put things, recognizes the places he/she has been before, knows how to go home, …remembers where he/she stores his/her things, …gets lost in familiar places, forgets how to go to a place that he/she has already been explained how to get to”.

### Procedure

The children voluntarily participated in this study with the written consent of their parents. The children who participated in this study were randomly assigned to one of two groups:

Group A. The group that played the ARSM task first and then answered the ARquestionnaire. Afterwards, they completed the AWMA Dot.

Group B. The group that completed the AWMA Dot first, and then played the ARSM task and completed the ARquestionnaire.

Both groups had a similar number of subjects. The participants were tested during two sessions (ARSM task and AWMA Dot) of approximately 45 minutes each, which generally took place on the same day. The testing took place Monday through Friday between 9:00 A.M. and 6:00 P.M. The parent written consent and the ECM-Q were returned before the testing session.

### Variables

For the analyses, we considered ten variables that are related to the performance in the ARSM task: duration of the experiment; blocks completed; trials completed; total trials; % trials passed; errors; approximation errors; % approximation errors; % of errors; and score. With regard to existing assessment approaches, we used the ECM-Q for the score of the ECM-Q questions. AWMA Dot refers to the Dot Matrix standardized score. For the ARquestionnaire, the satisfaction variable combines the answers of questions related to satisfaction, and usability combines the answers related to usability.

## Results

### ARSM task outcomes

**Figure 9:**
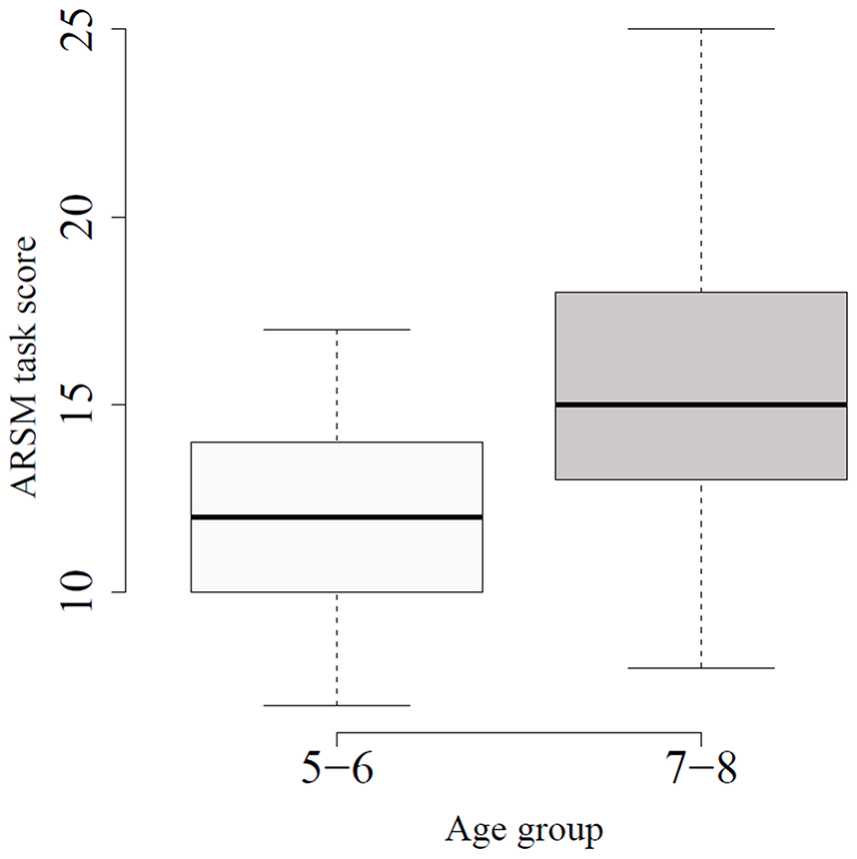
Boxplots of the score variable for the two age groups.

Several ANOVA tests were performed to determine if there were significant differences between the two age groups and for all the data stored during the execution of the ARSM task. We also provided the effect size. We used the Eta-squared (η^2^). The results can be observed in Table 2. Statistically significant differences were found in seven of the ten analyzed variables. The group of 7–8 year olds obtained significantly higher scores than the group of 5–6 year olds. This difference can also be observed in Figure 9. Moreover, the variable with the largest effect size was the ARSM task (see Table 2); however, another four variables also had a large effect size.

**Table 2:** ANOVA tests for the ARSM task variables (d.f. = 1, N = 76). The symbol ** indicates significant differences.

Variable	Group 5–6	Group 7–8	F-value	p-value	η^2^
Duration of the experiment (in minutes)	17.56±4.13	26.36±11.93	19.578	<0.001**	0.209
Blocks completed	1.83±0.44	2.63±0.84	27,917	<0.001**	0.274
Trials completed	7.20±1.66	9.71±2.78	23.735	<0.001**	0.242
Total trials	10.46±1.90	13.49±3.71	20.822	<0.001**	0.220
% trials passed	68.44±7.46	71.90±5.76	4.983	0.029**	0.063
Errors	3.27±0.92	3.77±1.31	3.835	0.054	0.049
Approximation errors	1.80±1.05	1.71±1.25	0.118	0.733	0.002
% approximation errors	55.49±26.46	43.84±27.76	3.500	0.065	0.045
% of errors	31.56±7.46	28.10±5.76	4.983	0.029**	0.063
Score	12.10±2.59	16.06±3.81	28.767	<0.001**	0.280

A multifactorial ANOVA test was also performed to take into consideration several factors simultaneously (Age Group and Gender). For the ARSM task score, the results showed that there was no statistically significant difference for the Gender factor (*F* [1] =  0.1699, *p* = 0.6814, η^2^  = 0.0023), or for the interaction between Age and Gender factors (F[1] = 0.034, *p* = 0.854, η^2^ = 0.0004). However, there were statistically significant differences for the Age Group factor (*F* [1] =  28.767, *p*<0.001**, η^2^ = 0.27993). These results can also be observed in Figure 9.

### AWMA Dot performance and ratings of the ECM-Q

**Figure 10:**
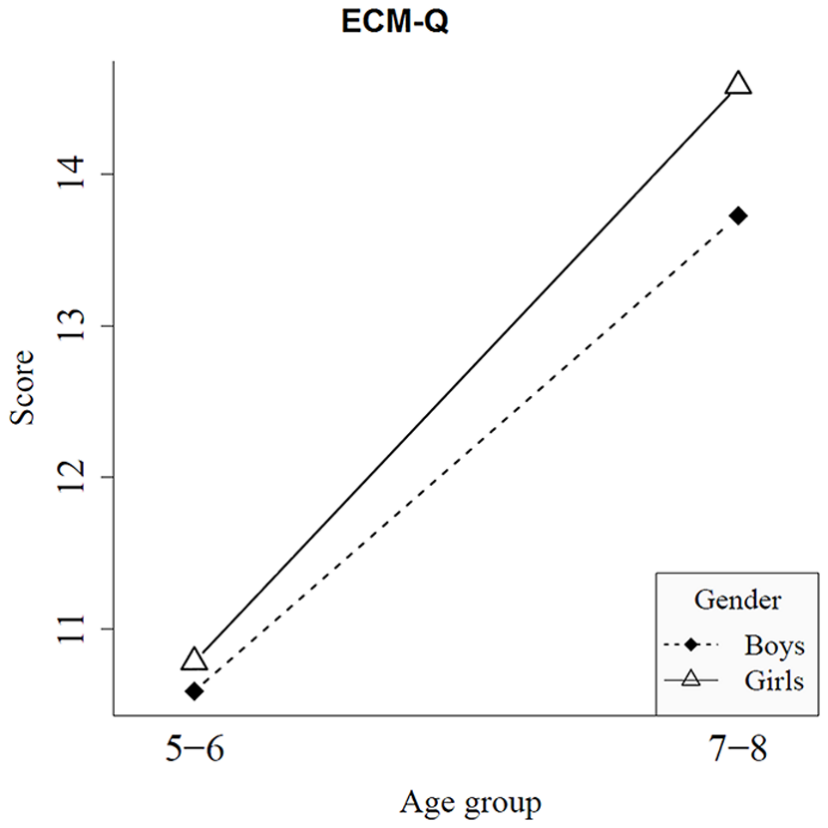
Traditional test (ECM-Q). Interaction by gender for the two age groups.

**Figure 11:**
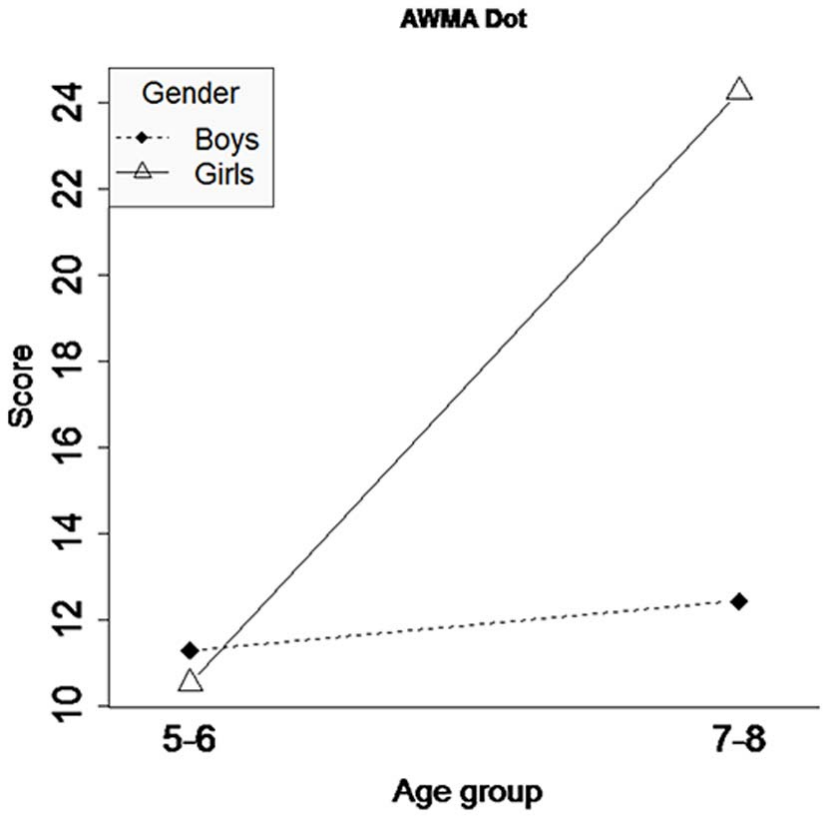
Traditional test (AWMA Dot). Interaction by gender for the two age groups.

Comparisons between the two age groups and the gender of the sample were done for the performance on the AWMA Dot and a parent questionnaire of the ECM-Q. Several ANOVA tests were performed to determine if there were significant differences between the group of 5–6 year olds and the group of 7–8 year olds (Table 3). The ARSM task score is also included in Table 3 to facilitate comparison. With regard to the traditional tests, the results show that no statistically significant differences were found. Another analysis considering gender is presented in Table 4. Figures 10 and 11 show the interaction plots for the traditional tests (ECM-Q and AWMA Dot) by Gender and Age Group. If these interaction plots are compared with the interaction plot of the ARSM task score, it can be observed that the most similar trend is offered by the parent questionnaires of the ECM-Q (Figure 10). AWMA Dot (Figure 11) presents a different trend.

**Table 3:** ANOVA tests for the traditional tests by Age Group (d.f. = 1, N = 76). The symbol ** indicates significant differences.

Variable	Group 5–6	Group 7–8	F-value	p-value	η^2^
ECM-Q	10.67±9.37	14.17±14.86	1.55	0.217	0.021
AWMA Dot	10.98±13.54	18.73±15.57	2.39	0.127	0.033
ARSM task score	12.10±2.59	16.06±3.81	28.77	<0.001**	0.280

**Table 4:** ANOVA tests for the traditional tests by gender (d.f. = 1, N = 76).

Variable	BOYS	GIRLS	F-value	p-value	η^2^
ECM-Q	11.89±11.40	12.74±12.98	1.33	0.253	0.018
AWMA Dot	11.71±15.83	17.18±25.29	1.19	0.279	0.017
ARSM task score	13.76±3.95	14.11±3.45	0.17	0.681	0.002

### Usability and satisfaction outcomes

The children answered 2 questions about usability and 6 questions about satisfaction. We checked to see if there were differences between the Age Group and Gender by performing two ANOVA tests for the individual questions (Tables 5 and 6) and for the two variables (Table 7). For the Age Group factor, statistically significant differences were found for only three questions (US#1, SA#5 and SA#6) in favor of the younger children. For the Gender factor, no statistically significant differences were found. For the usability and satisfaction variables, statistically significant differences were found only for the satisfaction variable in favor of the younger children. We would like to highlight the high values obtained for each question. These results show that the children were satisfied with the ARSM task and they thought it was easy to use.

**Table 5:** ANOVA tests for the individual questions grouped by age (d.f.  = 1, N = 76). The symbol ** indicates significant differences.

Question	Group 5–6	Group7–8	F-value	p-value	η^2^
SA#1: I have had a good time	4.87±0.33	4.80±0.47	0.57	0.451	0.007
US#1: I found the system easy to use	4.49±0.67	4.14±0.72	4.37	0.040**	0.057
US#2: I understood what I had to do at each moment (rules of the task)	4.77±0.53	4.80±0.40	0.07	0.783	0.001
SA#2: I liked the objects that appeared	4.67±0.69	4.60±0.60	0.19	0.664	0.002
SA#3: I liked that objects appeared inside the boxes	4.67±0.80	4.54±0.60	0.54	0.462	0.007
SA#4: I would recommend this system to my friends	4.46±0.78	4.11±0.78	3.54	0.063	0.046
SA#5: I would use this system again	4.51±0.90	4.06±0.89	4.62	0.034**	0.060
SA#6: Score the system from 1 to 5	4.92±0.27	4.69±0.46	7.25	0.008**	0.091

**Table 6:** ANOVA tests for the individual questions grouped by gender (d.f.  = 1, N = 76).

Question	Boys	Girls	*F*-value	*p*-value	η^2^
SA#1: I have had a good time	4.87±0.40	4.8±0.40	0.57	0.451	0.008
US#1: I found the system easy to use	4.38±0.74	4.26±0.69	0.56	0.453	0.008
US#2: I understood what I had to do at each moment (rules of the task)	4.87±0.33	4.69±0.57	2.89	0.093	0.039
SA#2: I liked the objects that appeared	4.72±0.55	4.54±0.73	1.33	0.252	0.018
SA#3: I liked that objects appeared inside the boxes	4.64±0.58	4.57±0.84	0.17	0.680	0.002
SA#4: I would recommend this system to my friends	4.31±0.76	4.29±0.85	0.01	0.907	<0.001
SA#5: I would use this system again	4.33±0.83	4.26±1.02	0.12	0.728	0.002
SA#6: Score the system from 1 to 5	4.79±0.4	4.83±0.38	0.13	0.716	0.002

**Table 7:** ANOVA tests for the satisfaction and usability variables (d.f.  = 1, N = 76). The symbol ** indicates significant differences.

Variable	Group 5_6	Group7_8	*F*-value	*p*-value	η^2^
SA: Satisfaction	4.68±0.69	4.47±0.71	10.53	0.001**	0.023
US: Usability	4.63±0.62	4.47±0.60	2.14	0.145	0.014

### Correlation outcomes

**Figure 12:**
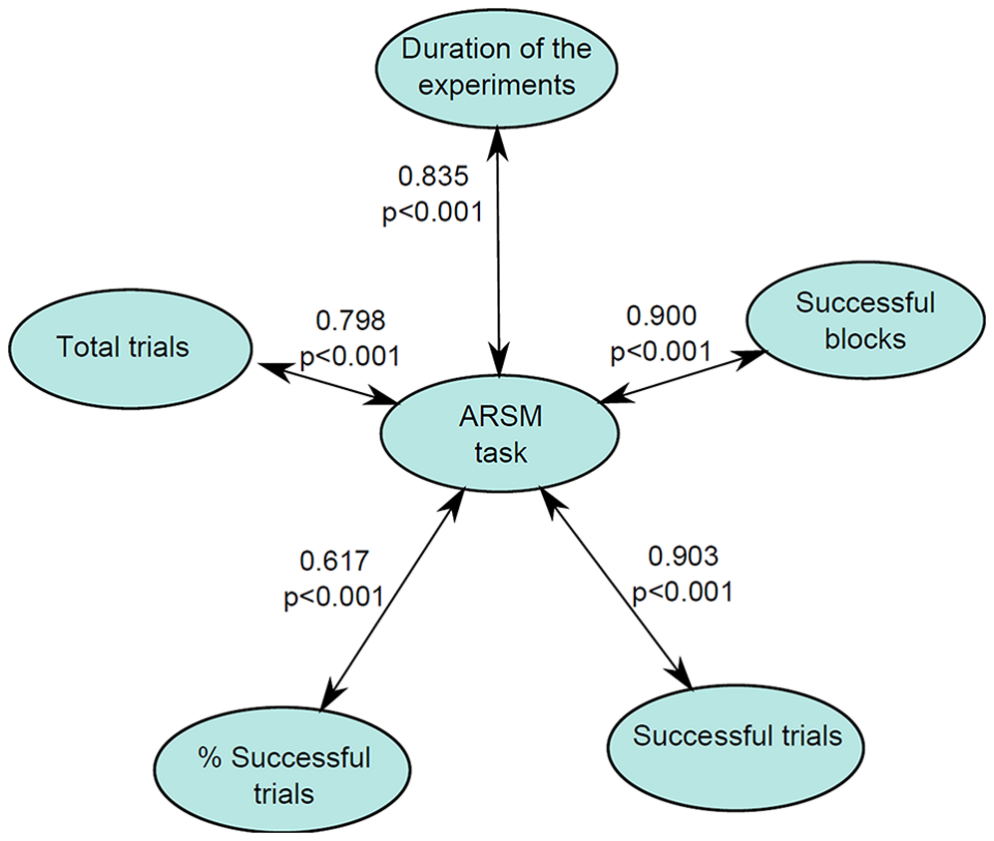
Correlations between the ARSM task score and the rest of the ARSM task variables.

An analysis was performed to determine if there were significant correlations between the ARSM task score and each of the remaining variables obtained from the task. These correlations are shown in Figure 12. Strong correlations were found between the task score and the following variables: duration of the experiments, total trials, successful blocks, successful trials, and % successful trials. These correlations indicate that the ARSM task score is an adequate overall measure. The ARSM task score also offers a significant correlation with the satisfaction variable (0.249, *p* = 0.014).

**Figure 13:**
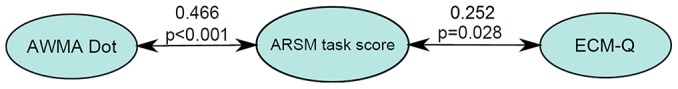
Correlations found between the ARSM task score and traditional tests.

To compare ARSM task performance's level with the performance level obtained in existing methods (AWMA Dot and ECM-Q), we calculated their correlations (Figure 13). These correlations indicate that the ARSM task score is correlated with all the methods used. However, the parent questionnaire of the ECM-Q is not correlated with AWMA Dot. Note that parent questionnaire of the ECM-Q and AWMA Dot are different approaches of spatial memory assessment.

## Discussion

In this study we tested the capability of our ARSM task to assess spatial short-term memory in 5–8 year olds. The designed instrument asked the participants to retain visuospatial information for a short period of time. The children had to remember the real place where visual objects were located, which were presented in AR. This novel instrument was compared with two different procedures commonly used in neuropsychological assessment: a standardized computerized tool (AWMA Dot, named Dot Matrix as a subtest of the AWMA battery for working memory assessment) and a parent's questionnaire about their child's spatial memory in everyday life. In addition, we evaluated its usability and perceived satisfaction.

There have been fewer attempts to address spatial short-term memory through experimental tasks involving the movement of the child around a real environment. The study of Smith et al. [36] presented a searching task for target locations that were hidden under a 7×7 grid. In this task, the child was trained to inspect each potential location and remember it to avoid revisiting locations that already had been inspected. In this task, the goal was simply to probe the search efficiency of the child. Piccardi et al. [37] tested the child's ability to retain several sequences of steps with spans of increasing difficulty in the Walking Corsi Test (WalCT). This test was a larger version of the Corsi Block-Tapping Test in which the child had to reproduce the walking sequence made by the experimenter in a surface area of 2.5 × 3 meters. Although the WalCT has some similarities with the ARSM task, our task involved an active role of the child. The participant had “a mission to accomplish” in order to help an animated character. In order to achieve the mission, the child was instructed to search for objects and remember where they were located. In the ARSM task, each spatial item had additional visual information and the number and distribution of locations varied between blocks. Hence, in the ARSM task the child had to remember all the visuospatial locations explored in a particular trial, whereas in the WalCT the child had to keep in mind the sequences of steps that another person had done.

With regard to the use of mobile AR for the assessment of spatial short-term memory, to our knowledge, this is the first time a system of these characteristics has been presented for this purpose. Nevertheless, mobile AR has already been proven to be an effective tool for other purposes such as learning different types of contents [28], [30]. The use of a mobile device allows the movement of the user in the real environment. This movement is a key factor for assessing the development of spatial orientation. The use of AR is also an important aspect that contributes to the potential of the ARSM task. The use of AR allows movement in a real environment and the appearance of virtual elements mixed in with the real environment. The advantages of AR over a Virtual Reality system are the following: first, with AR, the time and cost for developing the virtual scene is eliminated because the scene is the real one (i.e., a real maze); second, the participants can see their own body (e.g., hands or feet), whereas Virtual Reality only simulates this experience. Moreover, the children enjoy the AR experience (SA#3).

The ARSM task outcomes demonstrated age-related spatial memory improvement. The score and other indirect variables were significantly different between 5 and 8 years of age (Table 2 and Figure 9). It is well-known that visuospatial short-term memory skills increase as the brain develops [38], [39]. Therefore, our tool is designed to be sensitive to these maturational changes. In addition, we also consider that the ARSM task could be useful in the early detection of spatial orientation impairments that characterizes the developmental topographical disorientation syndrome [40], [41]. This syndrome has been related to the presence of certain minor neurological signs, such us difficulties in the formation of cognitive maps, poor sense of orientation and landmark recognition deficits [41]. Moreover, it has been shown that this improvement in visuospatial short-term memory has a relationship with abilities for mathematics [2] and for language mastery [4] that has repercussions in academic performance. In relation to this, some studies have shown a poorer performance on visuospatial span tasks in children with specific developmental disorders like dyscalculia [5], non-verbal learning disability [6], or specific language impairment [4]. Tasks of this kind assess the same process that was assessed in our novel task.

We did not find gender differences in ARSM task performance. It should be mentioned that there are contradictory results about this issue. Our findings support that boys do not outperform girls in short-term memory for object location, but it is difficult to establish comparisons with similar studies because this is the first time 5–8 year olds have been tested using this task. Conventional memory tests of visuospatial span in which the participant does not move have revealed that boys are superior [42]; however the object to remember in these tasks is very simple and does not vary between trials (e.g., dots). In addition, these types of tasks revealed that boys were smarter in pure spatial tasks and girls had better performance in verbal tasks [42]. It should be noted that when boys were asked to remember locations of common objects they did not outperform girls; however, the same task did show female superiority in adulthood [43]. This could suggest that both boys and girls might benefit from the features of our task, which requires spatial processing and object identification.

We compared children's performance in the ARSM task with the measures of the computerized AWMA Dot test. The AWMA Dot assesses the same cognitive process in stationary children and is part of a battery that has demonstrated a great capacity for detecting short-term memory failures that affect school activities [7], [44]. We found a significant correlation between the two measures (Figure 13). Furthermore, our task's results showed a larger correlation with the performance on the AWMA Dot than some of those calculated among the subtests of the AWMA battery. In addition, the size of our correlation was similar to that obtained between the AWMA Dot and measures of several traditional tests for short-term memory [45]. Even though our task involves large differences with the AWMA Dot in terms of the children's behaviour and stimuli used, the relation between the two tasks ensures that the ARSM task is able to assess the visuospatial short-term memory span.

With regard to the parent's ratings for their children's spatial memory, this type of measure showed a lower but significant correlation with the children's results in the ARSM task (Figure 13). The ECM-Q assesses the spatial memory performance of the children in real-world settings and involves the assessment of spatial short-term and long-term memories. This measure not only reflects the cognitive process, but it also reflects the ability of the boy or girl to competently use the skill in everyday life. It has been proposed that the correlation between measures of a traditional cognitive test and scores on these questionnaires demonstrates the capability of a test to assess the performance of the person with ecological validity [26]. Our results nicely report that the performance in the ARSM task reflected the level of visuospatial short-term memory span as well as the performance on everyday tasks that require spatial memory skill. In addition, the performance in the AWMA Dot did not correlate with scores in the ECM-Q. The absence of a relation between the two measures could be due to the fact that the ECM-Q also involved spatial long-term memory, which is not assessed in the AWMA Dot test. However, it could also show the low similarity with real-world functioning. Nevertheless, altogether the correlations between scores in the ARSM task and the traditional test demonstrated the verisimilitude of the ARSM task with a spatial short-term task imposed in an everyday environment.

With regard to the usability, several authors have considered usability to be an important factor that affects educational effectiveness [46]–[48]. Sun et al. [49] argued that systems that are easy to use help students to focus their attention on the content. In our case, the ARSM task was easy to manipulate (with means above 4 on a scale of 1 to 5 for the two usability questions). In addition, the people observing the participants during the task stated that a great majority of users did not have any problems interacting with the device. Therefore, based on these considerations, our ARSM task does help students to focus their attention on the task content.

With regard to the satisfaction and its relationship with learning outcomes, several previous works have analyzed this relationship. For example, in an online course, Lee et al. [50] found a correlation between satisfaction and learning outcomes. In our case, we have also found a correlation between the satisfaction variable and the ARSM task score. This indicates that perceived satisfaction is related to the ARSM task score. In our study, there was an interesting result concerning the age difference in perceived satisfaction (Table 7). The younger children reported higher satisfaction with the ARSM task experience. Even though the values for the two groups are very high, this higher value could be related to the length of time they were playing. The 5–6 year olds played less time because they did not pass the more difficult task blocks. This may be the reason why the younger children perceived the system as being easier to use (Table 5, question US#1). Therefore, the task could be perceived as less boring and difficult at these ages. Also, the 7–8 year olds could have felt less comfortable doing the task because they were more conscious about their errors, which is in line with the developmental changes in metacognition [51].

## Conclusions

We have developed the first AR task to assess spatial short-term memory in children. A preliminary study determined the most appropriate size of the device for the ARSM task. The tasks assessed the ability of a child to retain real-world locations of an increasing number of objects that appeared in AR. We compared the ARSM task's performance with traditional neuropsychological procedures and we measured the usability and satisfaction of the participants for the ARSM task. The performance in the ARSM task showed normal age-related short-term memory improvement for children 5–8 years of age. Also, the ARSM task demonstrated similitude with everyday spatial memory activities and with a traditional measure of visuospatial short-term memory. The ARSM task could be used as an entertaining method to assess or train children in spatial short-term memory skills. However, to corroborate this statement, another study would need to be conducted. As other future work, we would like to study the power of the ARSM task to detect learning difficulties in samples of people with academic problems or neurological disorders. In addition, it could be interesting to compare the performance on the ARSM task with the performance on other spatial tasks that require navigation on the real world (e.g. [37]). The possibilities of our task for adults could also be studied in future works.

## Acknowledgments

- We would like to thank the following for their contributions:

▪ The “Escola d′Estiu” and especially Juan Cano, Miguelón Giménez, and Javier Irimia. This work would not have been possible without their collaboration.

▪ Our thanks to Alejandro del Río, Minerva Rodríguez and Lucía Cubel for their help during the development and to the children's parents who signed the agreement to allow their children to participate in the study and who filled out the parent's questionnaires.

▪ The children who participated in the study.

▪ The ETSInf for letting us use its facilities during the testing phase.

▪ The reviewers for their valuable suggestions.

## Supporting Information

Data S1 **Raw data file. Data used in the analysis.**
(XLS)

## References

[1] Linn MC, Petersen AC (1985) Emergence and characterization of gender differences in spatial abilities: a meta-analysis. Child Development 56:1479–1498. 4075870

[2] Simmons FR, Willis C, Adams A-M (2012) Different components of working memory have different relationships with different mathematical skills. Journal of experimental child psychology 111:139–155 10.1016/j.jecp.2011.08.011 22018889

[3] Alloway TP, Alloway RG (2010) Investigating the predictive roles of working memory and IQ in academic attainment. Journal of experimental child psychology 106:20–29 10.1016/j.jecp.2009.11.003 20018296

[4] Bavin EL, Wilson PH, Maruff P, Sleeman F (2005) Spatio-visual memory of children with specific language impairment: evidence for generalized processing problems. International journal of language & communication disorders 40:319–332 10.1080/13682820400027750 16195191

[5] Szucs D, Devine A, Soltesz F, Nobes A, Gabriel F (2013) Developmental dyscalculia is related to visuo-spatial memory and inhibition impairment. Cortex; a journal devoted to the study of the nervous system and behavior 49:2674–2688 10.1016/j.cortex.2013.06.007 23890692 PMC3878850

[6] Mammarella IC, Cornoldi C (2014) An analysis of the criteria used to diagnose children with Nonverbal Learning Disability (NLD). Child neuropsychology: a journal on normal and abnormal development in childhood and adolescence 20:255–280 10.1080/09297049.2013.796920 23705673

[7] Alloway TP (2007) Automated Working Memory Assessment. London: The Psychological Corporation.

[8] Oades RD, Isaacson RL (1978) The development of food search behavior by rats: The effects of hippocampal damage and haloperidol. Behavioral Biology 40:327–337 10.1016/S0091-6773(79)90184-6 743067

[9] Morris R (1984) Developments of a water-maze procedure for studying spatial learning in the rat. Journal of Neuroscience Methods 11:47–60 10.1016/0165-0270(84)90007-4 6471907

[10] Olton DS (1987) The radial arm maze as a tool in behavioral pharmacology. Physiology & Behavior 40:793–797 10.1016/0031-9384(87)90286-1 3313453

[11] Méndez-López M, Méndez M, López L, Arias JL (2009) Sexually dimorphic c-Fos expression following spatial working memory in young and adult rats. Physiology & behavior 98:793–797 10.1016/j.physbeh.2009.06.006 19545582

[12] Munoz M, Morris RGM (2009) Episodic memory in animals. In:Squire LR editor. New Encyclopedia of Neuroscience. Oxford: Academic Press. pp.1173–1182.

[13] Shore DI, Stanford L, Macinnes WJ, Klein RM, Brown RE (2001) Of mice and men: Virtual Hebb-Williams mazes permit comparison of spatial learning across species. Cognitive, Affective, & Behavioral Neuroscience 1:83–89 10.3758/CABN.1.1.83 12467105

[14] Astur RS, Taylor LB, Mamelak AN, Philpott L, Sutherland RJ (2002) Humans with hippocampus damage display severe spatial memory impairments in a virtual Morris water task. Behavioural Brain Research 132:77–84 10.1016/S0166-4328(01)00399-0 11853860

[15] Astur RS, Tropp J, Sava S, Constable RT, Markus EJ (2004) Sex differences and correlations in a virtual Morris water task, a virtual radial arm maze, and mental rotation. Behavioural brain research 151:103–115 10.1016/j.bbr.2003.08.024 15084426

[16] Kelly DM, Gibson BM (2007) Spatial navigation: Spatial learning in real and virtual environments. Comparative Cognition & Behavior Reviews 2:111–124 10.3819/ccbr.2008.20007

[17] Sturz BR, Bodily KD (2010) Encoding of variability of landmark-based spatial information. Psychological research 74:560–567 10.1007/s00426-010-0277-4 20177902

[18] Cánovas R, García RF, Cimadevilla JM (2011) Effect of reference frames and number of cues available on the spatial orientation of males and females in a virtual memory task. Behavioural brain research 216:116–121 10.1016/j.bbr.2010.07.026 20655953

[19] Cimadevilla JM, Cánovas R, Iribarne L, Soria A, López L (2011) A virtual-based task to assess place avoidance in humans. Journal of neuroscience methods 196:45–50 10.1016/j.jneumeth.2010.12.026 21219930

[20] Cheng K (1986) A purely geometric module in the rat's spatial representation. Cognition 23:149–178 10.1016/0010-0277(86)90041-7 3742991

[21] Burgess N, Maguire EA, Spiers HJ, O′Keefe J (2001) A temporoparietal and prefrontal network for retrieving the spatial context of lifelike events. NeuroImage 14:439–453 10.1006/nimg.2001.0806 11467917

[22] Burgess N, Maguire EA, O′Keefe J (2002) The Human Hippocampus and Spatial and Episodic Memory. Neuron 35:625–641 10.1016/S0896-6273(02)00830-9 12194864

[23] Koening ST, Crucian GP, Dünser A, Bartneck C, Dalrymple-Alford JC (2011) Validity evaluation of a spatial memory task in virtual environments. International Journal of Design and Innovation Research 6:1–13.

[24] Passolunghi MC, Mammarella IC (2012) Selective spatial working memory impairment in a group of children with mathematics learning disabilities and poor problem-solving skills. Journal of learning disabilities 45:341–350 10.1177/0022219411400746 21444930

[25] Thomas E, Reeve R, Fredrickson A, Maruff P (2011) Spatial memory and executive functions in children. Child neuropsychology 17:599–615 10.1080/09297049.2011.567980 21557119

[26] Spooner DM, Pachana NA (2011) Ecological validity in neuropsychological assessment: a case for greater consideration in research with neurologically intact populations. Archives of clinical neuropsychology 21:327–337 10.1016/j.acn.2006.04.004 16769198

[27] Juan MC, Alcañiz M, Monserrat C, Botella C, Banos RM, et al (2005) Using Augmented Reality to Treat Phobias. IEEE Computer Graphics and Applications 25:31–37 10.1109/MCG.2005.143 16315475

[28] Furió D, González-Gancedo S, Juan MC, Seguí I, Costa M (2013) The effects of the size and weight of a mobile device on an educational game. Computers & Education 64:24–41.

[29] Juan MC, Furió D, Alem L, Ashworth P, Cano J (2011) ARGreenet and BasicGreenet: Two mobile games for learning how to recycle. Proceedings of the 19th International Conference on Computer Graphics, Visualization and Computer Vision. pp.25–32.

[30] Furió D, González-Gancedo S, Juan MC, Seguí I, Rando N (2013) Evaluation of learning outcomes using an educational iPhone game vs. traditional game. Computers & Education 64:1–23.

[31] Albrecht U-V, Folta-Schoofs K, Behrends M, von Jan U (2013) Effects of mobile augmented reality learning compared to textbook learning on medical students: randomized controlled pilot study. Journal of medical Internet research 15:e182 10.2196/jmir.2497 23963306 PMC3758026

[32] Liu P-HE, Tsai M-K (2013) Using augmented-reality-based mobile learning material in EFL English composition: An exploratory case study. British Journal of Educational Technology 44:E1–E4 10.1111/j.1467-8535.2012.01302.x

[33] Baddeley AD (1986) Working memory. Oxford: Clarendon Press.

[34] Alloway TP (2012) Working Memory Assessment. Second Edi. London: Pearson Assessment.

[35] Kamphaus KW, Perez-Hernandez E, Sanchez-Sanchez F (2014) Cuestionario de Evaluación Clínica de la Memoria. In press. Madrid: TEA Ediciones.

[36] Smith AD, Gilchrist ID, Hood BM (2005) Children's search behaviour in large-scale space: developmental components of exploration. Perception 34:1221–1229 Available: http://www.ncbi.nlm.nih.gov/pubmed/16309116. Accessed 2014 September 26.. 16309116 10.1068/p5270

[37] Piccardi L, Palermo L, Leonzi M, Risetti M, Zompanti L, et al (2014) The Walking Corsi Test (WalCT): a normative study of topographical working memory in a sample of 4- to 11-year-olds. The Clinical neuropsychologist 28:84–96 10.1080/13854046.2013.863976 24580053

[38] Gathercole SE, Pickering SJ, Ambridge B, Wearing H (2004) The Structure of Working Memory From 4 to 15 Years of Age. Developmental Psychology 40:177–190 10.1037/0012-1649.40.2.177 14979759

[39] Best JR, Miller PH (n.d.) A developmental perspective on executive function. Child development 81:1641–1660 10.1111/j.1467-8624.2010.01499.x PMC3058827 21077853

[40] Bianchini F, Incoccia C, Palermo L, Piccardi L, Zompanti L, et al (2010) Developmental topographical disorientation in a healthy subject. Neuropsychologia 48:1563–1573 10.1016/j.neuropsychologia.2010.01.025 20144632

[41] Iaria G, Barton JJS (2010) Developmental Topographical Disorientation: a newly discovered cognitive disorder. Experimental brain research 206:189–196 Available: http://www.ncbi.nlm.nih.gov/pubmed/20431873. Accessed 2014 September 26.. 20431873 10.1007/s00221-010-2256-9

[42] Lowe PA, Mayfield JW, Reynolds CR (2003) Gender differences in memory test performance among children and adolescents. Archives of Clinical Neuropsychology 18:865–878 10.1093/arclin/18.8.865 14609581

[43] Barnfield AM (1999) Development of sex differences in spatial memory. Perceptual and Motor Skills 89:339–350 10.2466/pms.1999.89.1.339 10544436

[44] Alloway TP, Gathercole SE, Kirkwood H, Elliott J (2009) The working memory rating scale: A classroom-based behavioral assessment of working memory. Learning and Individual Differences 19:242–245 10.1016/j.lindif.2008.10.003

[45] Injoque-Ricle I, Calero AD, Alloway TP, Burin DI (2011) Assessing working memory in Spanish-speaking children: Automated Working Memory Assessment battery adaptation. Learning and Individual Differences 21:78–84 10.1016/j.lindif.2010.09.012

[46] Jones A, Scanlon E, Tosunoglu C, Morris E, Ross S, et al (1999) Contexts for evaluating educational software. Interacting with Computers 11:499–516.

[47] Mayes JT, Fowler CJ (1999) Learning technology and usability: A framework for understanding courseware. Interacting with Computers 11:485–497.

[48] Squires D, Preece J (1999) Predicting quality in educational software. Interacting with Computers 11:467–483 10.1016/S0953-5438(98)00063-0

[49] Sun PC, Tsai RJ, Finger G, Chen YY, Yeh D (2008) What drives a successful e-Learning? An empirical investigation of the critical factors influencing learner satisfaction. Computers & Education 50:1183–1202.

[50] Lee SJ, Srinivasan S, Trail T, Lewis D, Lopez S (2011) Examining the relationship among student perception of support, course satisfaction, and learning outcomes in online learning. The Internet and Higher Education 14:158–163 10.1016/j.iheduc.2011.04.001

[51] Lyons KE, Zelazo PD (2011) Monitoring, metacognition, and executive function: elucidating the role of self-reflection in the development of self-regulation. In:Benson Jeditor. Advances in Child Development and Behavior. Burlington: Academic Press. pp.379–412. 10.1016/b978-0-12-386491-8.00010-4 21887967

